# CD133^+^CD54^+^CD44^+^ circulating tumor cells as a biomarker of treatment selection and liver metastasis in patients with colorectal cancer

**DOI:** 10.18632/oncotarget.12675

**Published:** 2016-10-15

**Authors:** Chao Fang, Chuanwen Fan, Cun Wang, Qiaorong Huang, Wentong Meng, Yongyang Yu, Lie Yang, Zhihai Peng, Jiankun Hu, Yuan Li, Xianming Mo, Zongguang Zhou

**Affiliations:** ^1^ Department of Gastrointestinal Surgery, West China Hospital, Sichuan University, Chengdu, China; ^2^ Institute of Digestive Surgery, State Key Laboratory of Biotherapy, West China Hospital, Sichuan University, Chengdu, China; ^3^ Laboratory of Stem Cell Biology, State Key Laboratory of Biotherapy, West China Hospital, Sichuan University, Chengdu, China; ^4^ Department of General Surgery, Shanghai First People's Hospital, Shanghai Jiaotong University, Shanghai, China

**Keywords:** colorectal cancer, circulating tumor cells, CD133^+^CD54^+^CD44^+^ cellular subpopulation, liver metastasis, predictive biomarker

## Abstract

**Introduction:**

Liver is the most common site of distant metastasis in colorectal cancer (CRC). Early diagnosis and appropriate treatment selection decides overall prognosis of patients. However, current diagnostic measures were basically imaging but not functional. Circulating tumor cells (CTCs) known as hold the key to understand the biology of metastatic mechanism provide a novel and auxiliary diagnostic strategy for CRC with liver metastasis (CRC-LM).

**Results:**

The expression of CD133^+^ and CD133^+^CD54^+^CD44^+^ cellular subpopulations were higher in the peripheral blood of CRC-LM patients when compared with those without metastasis (*P*<0.001). Multivariate analysis proved the association between the expression of CD133^+^CD44^+^CD54^+^ cellular subpopulation and the existence of CRC-LM (*P*<0.001). The combination of abdominal CT/MRI, CEA and the CD133^+^CD44^+^CD54^+^ cellular subpopulation showed increased detection and discrimination rate for liver metastasis, with a sensitivity of 88.2% and a specificity of 92.4%. Meanwhile, it also show accurate predictive value for liver metastasis (OR=2.898, 95% C.I.1.374–6.110).

**Materials and Method:**

Flow cytometry and multivariate analysis was performed to detect the expression of cancer initiating cells the correlation between cellular subpopulations and liver metastasis in patients with CRC. The receiver operating characteristic curves combined with the area under the curve were generated to compare the predictive ability of the cellular subpopulation for liver metastasis with current CT and MRI images.

**Conclusions:**

The identification, expression and application of CTC subpopulations will provide an ideal cellular predictive marker for CRC liver metastasis and a potential marker for further investigation.

## INTRODUCTION

One of the critical prognostic factors for CRC is the existence of liver metastasis and approximately 50% to 60% of CRC patients will develop synchronous or metachronous liver metastasis sooner or later [1-5]. Thus, the clinical evaluation of synchronous liver metastasis is necessary to determine an effective treatment strategy thus ultimately improve the survival of patients with CRC [2]. Currently, the diagnosis of liver metastasis mainly based on CT and MRI images. However, these methods have limited value in precise diagnosis of early progression of liver metastasis or differential diagnosis from benign nodules of liver. Therefore, it is imperative to identify novel functional biomarkers in its diagnosis.

Tumor cells circulating in the blood stream are referred as circulating tumor cells (CTCs) [6] while a fraction of these CTCs are known as metastasis-initiating cells (MICs) [7]. The MICs hold the key to understand the biology of metastatic mechanism while also served as novel biomarkers of noninvasively measuring of tumor genotypes. The emergence of increasingly advanced and sensitive technologies to measure and isolate CTCs provides the opportunity to study these MICs in detail. Current approaches in cell selection mainly rely on those physical properties, expression of cell surface biomarkers, or functional characteristics of CTCs [6-8]. For example, the CellSearch System by Veridox enumerates those epithelial cell adhesion molecule (EPCAM) expressing carcinoma cells [9]. Nevertheless, recent studies have demonstrated that the disseminating carcinoma cells may undergo kind of epithelial-to-mesenchymal transition (EMT), which may result in at least partial down regulation or even loss of epithelium-specific molecules [10-12]. Thus, the use of the epithelial antigen EPCAM as a selection marker may not be an optimal choice due to its low sensitivity with a median yield of approximately one CTC per milliliter [11,12].

To circumvent these limitations, additional methods to detect CTC surface markers have been developed, including flow cytometry in the form of fluorescence-activated cell sorting (FACS) [6,13]. Furthermore, an abundance of cell surface markers, such as CD133 [14,15], CD44 [15,16], CD26 [17,18], CD24 [19], CD166 [20,21], have been reported to be useful in detection and identification of tumor cells, cancer initiating cells and CTCs, in breast, prostate, lung, colon, rectum, and other solid tumors. Previously, we found that the rare CD44^+^CD54^+^ cellular subpopulation in rectal [22] and gastric cancer tissues [23] can potentially identify the early progression of cancer. Here in this study, we aimed to investigate and identify new cell surface markers or their combinations that could be used as the baseline to measure CTC in the peripheral blood of patients with CRC. We hypothesized that the cellular subpopulations of CTCs in the peripheral blood have the potential of predicting liver metastasis in patients with CRC.

## RESULTS

### Cellular subpopulation with stable CD133^+^ expression could be used as the baseline measurement for peripheral blood ctcs in patients with CRC

**Figure 1:**
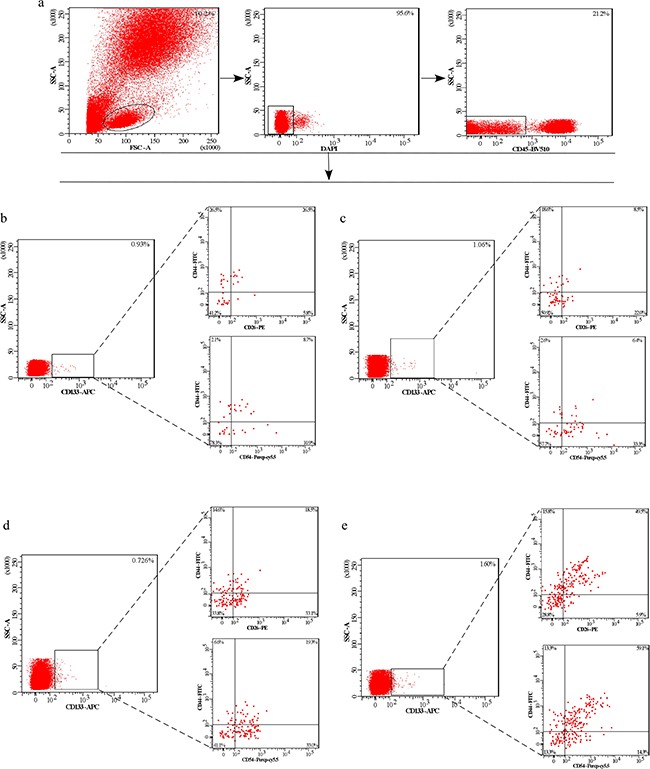
The expression of CD133^+^, CD133^+^CD54^+^CD44^+^ and CD133^+^CD26^+^CD44^+^ cellular subpopulation in the peripheral blood of CRC patients and individuals **a.** Left. Gating strategy to detect cellular subpopulation in whole blood or blood depleted of hematopoietic cells by FACS. Middle and right. Gating strategy to select DAPI^−^CD45^−^ cells. **b - e.** Plots are gated on DAPI^−^CD45^−^ cells. Contour plots show the expression of CD133^+^, CD133^+^CD54^+^CD44^+^ and CD133^+^CD26^+^CD44^+^ cellular subpopulation in the peripheral blood of CRC patients and health individuals. Percentages of cells are indicated for each gate or plot quadrant. (b, c, d, e) Left. The expression of CD133^+^ cellular subpopulation. (b, c, d, e) Low right. The expression of CD133^+^CD54^+^CD44^+^ cellular subpopulation. Right upper. The expression of CD133^+^CD26^+^CD44^+^ cellular subpopulation. (b) Peripheral blood of health individual. (c) Peripheral blood of early CRC patient (Dukes I/II). (d) Peripheral blood of CRC patient with lymph node metastasis (Dukes III). (e) Peripheral blood of CRC patient with liver metastasis (Dukes IV).

Firstly, we collected 20 peripheral blood samples from 10 CRC patients and 10 age-matched healthy controls. The expression of thirteen cell surface markers, associated with epithelial cancers or poor prognosis were measured and compared between the two groups (Supplementary Table S1). Stable expression of CD26^+^, CD44^+^, CD54^+^, CD133^+^, EPCAM^+^ was demonstrated in the peripheral blood of both CRC and healthy controls by flow cytometry. Furthermore, compared with control, the expression of CD133^+^ was significantly higher in the CRC group (Supplementary Table S1, Figure 1). As showed in Figure 1, CD133^+^ cellular subpopulation could easily be identified from CD133^−^ cellular subpopulation by flow cytometry. These data suggested an enrichment of CD133^+^ cellular subpopulation in CRC patients while the CD133^+^ cellular subpopulation could be used as the baseline measurement for CTCs in the peripheral blood of CRC patients in FACS analysis.

### Identification of CD133^+^ Based CTC subpopulations in the peripheral blood of patients with CRC

**Figure 2:**
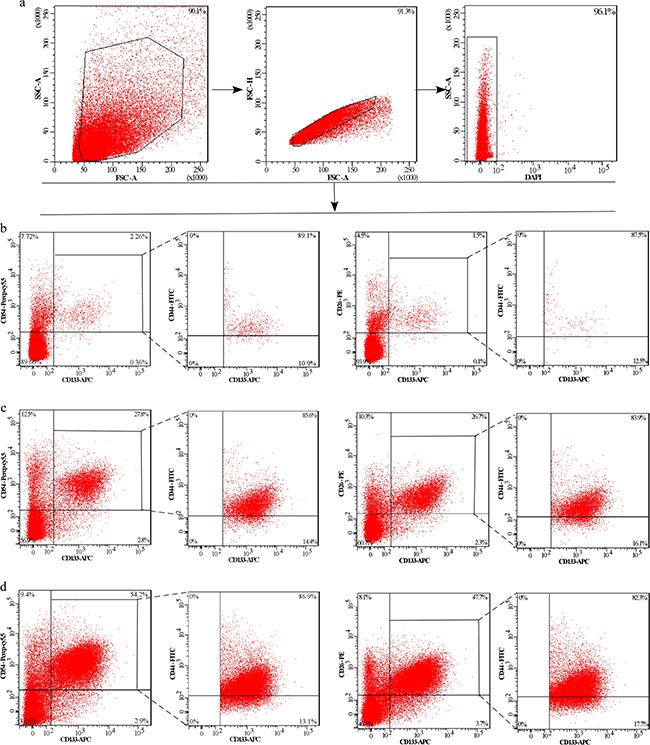
The expression of CD133^+^CD54^+^, CD133^+^CD26^+^, CD133^+^CD54^+^CD44^+^ and CD133^+^CD26^+^CD44^+^ cellular subpopulation in the cells suspension of tissues **a.** Gating strategies to select DAPI^−^ cells in the cells suspension. Plots are gated on the DAPI^−^ cells. Contour plots show the expression of CD133^+^CD54^+^, CD133^+^CD26^+^, CD133^+^CD54^+^CD44^+^ and CD133^+^CD26^+^CD44^+^ cellular subpopulation in the cells suspension of CRC liver metastases, CRC primary tissue and corresponding normal tissues. **b.** Cells suspension of corresponding normal tissue. **c.** Cells suspension of CRC primary tissue. **d.** Cells suspension of CRC liver metastases.

Next, we measured the co-expression of three biomarkers to screen the potential biomarkers of metastasis-related in CRC. The first combination is CD133, CD54 and CD44, and the second combination is CD133, CD26 and CD44. Twenty peripheral blood samples were collected from five healthy controls, five patients with early stage CRC, five with N+ CRC (CRC-LN), and five with CRC and liver metastasis (CRC-LM)). Flow cytometry analysis found that the co-expression of both the above 2 combinations was higher in the CRC-LM group when compared with the other three groups. However, no significant difference was observed between the other three groups (Figure 1b, 1c, 1d, and 1e). In addition, the expression and co-expression of CD133, CD44, CD54 and CD26 were also examined in the cells suspension derived from fresh tissue of colorectal cancer and liver metastases. We found that the expression of the CD133^+^CD54^+^, CD133^+^CD26^+^, CD133^+^CD54^+^CD44^+^, CD133^+^CD26^+^CD44^+^cellular subpopulation was significantly higher in liver tissues (Figure 2b, 2c, and 2d). These data indicated that cells expressing CD133^+^CD54^+^CD44^+^ and CD133^+^CD26^+^CD44^+^ in the peripheral blood were associated with liver metastasis.

### The relationship between clinicopathological characteristics and CTC subpopulations

**Figure 3:**
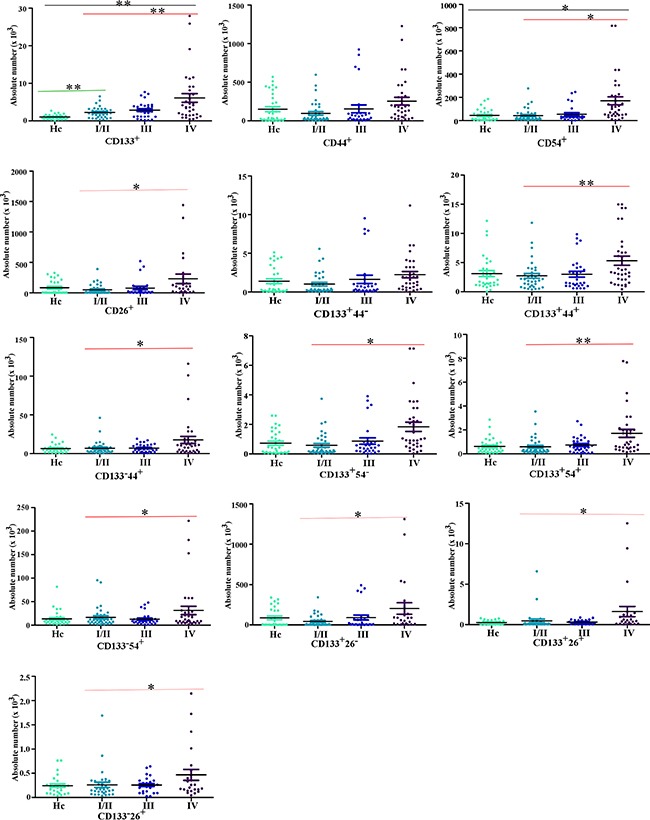
The comparison of the expression of cellular subpopulations in the peripheral blood of CRC patients and health control (Hc) The comparison of cellular subpopulations (the expression of CD133, CD44, CD26 and CD54 respectively or two markers federatively) in the peripheral blood of CRC patients and health control. As Hc for the health individuals, I/II for CRC patients with Dukes I/II (early CRC), III for CRC patients with Dukes III (CRC patients with lymph node metastasis), IV for CRC patients with Dukes IV (CRC patients with liver metastasis). (**P<0.001, *P<0.05).

**Figure 4:**
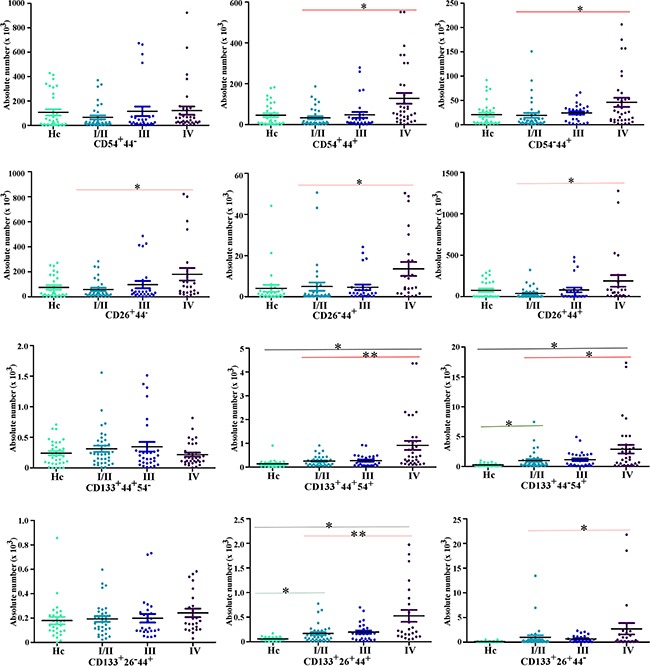
The comparison of the expression of cellular subpopulations in the peripheral blood of CRC patients and health control (Hc) The comparison of cellular subpopulations (the expression of CD133, CD44, CD26 and CD54, two or three markers federatively) in the peripheral blood of CRC patients and health control. As Hc for the health individuals, I/II for CRC patients with Dukes I/II (early CRC), III for CRC patients with Dukes III (CRC patients with lymph node metastasis), IV for CRC patients with Dukes IV (CRC patients with liver metastasis). (**P<0.001, *P<0.05).

Then, we collected 100 peripheral blood samples from CRC patients, including 36 patients with early stage CRC, 30 CRC-LN patients and 34 CRC-LM patients. In addition, we also collected 33 peripheral blood samples from healthy individuals. The relationship between clinicopathological characteristics of included patients and CTC subpopulations was summarized in Table 1 and Figure 3, 4. We found that liver metastasis is significantly associated with serum CEA levels (*P*<0.001), serum CA19-9 levels (*P* <0.001), extra nodal tumor deposits (*P*<0.001), CD133^+^ subpopulation (*P* <0.001), CD133^+^CD44^+^ subpopulation (*P*=0.001), CD133^+^CD54^+^ subpopulation (*P*<0.001), CD133^+^CD44^+^CD26^+^ subpopulation (*P*<0.001) and CD133^+^CD44^+^CD54^+^ subpopulation (*P*<0.001). No clinicopathological characteristics or cellular subpopulations of CTCs found to be associated with lymph node metastasis (Table 1, Figure 3 and 4.). Furthermore, we compared the cellular subpopulations of CTCs between health individuals and CRC patients. The results also indicated that the relationship between CRC and CD133^+^ subpopulation (*P*<0.001), CD54^+^ subpopulation (*P*=0.046), CD133^+^CD44^+^CD54^+^ subpopulation (*P*=0.003), CD133^+^CD44^+^CD54^+^ subpopulation (*P*=0.005), and CD133^+^CD44^−^CD54^+^ subpopulation (*P*=0.005) (Supplementary Table S2, Figure 3, 4.). No cellular subpopulation of CTCs found to be associated with early CRC (Supplementary Table S2, Figure 3, 4.).

**Table 1:** Clinical characteristics and cellular subpopulations of CTCs of CRC

	Liver metastasis		*P* value	Lymph node metastasis		*P* value
	No (n)	Yes (n)		No (n)	Yes (n)	
Gender (male: female)	44:22	18:16	0.180	24:12	20:10	1
Age (years)	62.85±10.47	58.32±11.79	0.053	63.78±11.85	61.73±8.58	0.434
Locations			0.337			0.011
Rectum	47	21		21	26	
Colon	19	13		15	4	
Serum CEA level^a^ (n)			<0.001			0.295
0	43	5		26	17	
1	9	10		6	4	
2	11	14		3	7	
3	3	5		1	2	
Serum CA19-9 level^b^ (n)			<0.001			0.336
0	47	15		28	20	
1	12	8		6	5	
2	6	3		2	5	
3	0	8		0	0	
Extra nodal tumor deposits			<0.001			0.051
Absent	60	14		35	25	
Present	6	20		1	5	
CD133^+^ subpopulation (×10^3^)	2.81±0.23	6.12±0.67	<0.001	2.78±0.26	2.83±0.21	0.920
CD54^+^ subpopulation (×10^3^)	136.80±26.29	340.12±40.15	0.003	42.97±11.26	100.02±18.42	0.082
CD26^+^ subpopulation (×10^3^)	69.85±12.48	238.77±40.69	0.005	50.29±8.61	94.68±15.97	0.177
CD44^+^ subpopulation (×10^3^)	183.87±40.69	254.37±68.80	0.370	95.50±14.04	289.91±57.10	0.053
CD133^+^CD44^−^ subpopulation (×10^3^)	1.87±0.39	2.48±0.57	0.427	0.99±0.13	1.47±0.27	0.047
CD133^+^CD44^+^ subpopulation(×10^3^)	0.57±0.11	1.07±0.45	0.001	0.54±0.12	0.61±0.13	0.628
CD133^−^CD44^+^ subpopulation (×10^3^)	7.54±0.72	17.64±2.67	0.003	6.99±0.86	7.12±0.53	0.947
CD133^+^CD54^−^ subpopulation (×10^3^)	0.97±0.18	1.84±0.19	0.023	1.16±0.19	1.42±0.24	0.054
CD133^+^CD54^+^ subpopulation (×10^3^)	0.65±0.07	1.71±0.19	<0.001	0.58±0.14	0.73±0.17	0.378
CD133^−^CD54^+^ subpopulation (×10^3^)	1.48±0.17	3.13±0.52	0.021	1.66±0.21	1.27±0.13	0.379
CD133^+^CD26^−^ subpopulation (×10^3^)	0.26±0.03	0.47±0.05	0.022	0.26±0.03	0.26±0.02	0.977
CD133^+^CD26^+^ subpopulation (×10^3^)	0.38±0.09	1.61±0.31	0.007	0.45±0.12	0.30±0.03	0.529
CD133^−^CD26^+^ subpopulation (×10^3^)	64.65±12.14	210.81±37.03	0.008	43.27±7.58	91.79±15.96	0.129
CD26^+^CD44^−^ subpopulation (×10^3^)	36.71±8.86	128.90±24.50	0.012	38.86±5.60	69.38±12.56	0.195
CD26^+^CD44^+^ subpopulation (×10^3^)	25.79±6.31	94.63±36.21	0.010	17.38±4.21	43.89±8.12	0.118
CD26^−^CD44^+^ subpopulation (×10^3^)	4.76±0.98	13.47±1.65	0.004	4.87±1.15	4.61±0.73	0.919
CD54^+^CD44^−^ subpopulation (×10^3^)	61.47±12.78	121.80±19.56	0.057	65.86±9.87	193.64±35.29	0.042
CD54^+^CD44^+^ subpopulation (×10^3^)	63.58±14.81	128.45±15.29	0.043	33.49±4.59	99.69±21.01	0.070
CD54^−^CD44^+^ subpopulation (×10^3^)	23.92±2.90	46.16±5.49	0.039	19.24±3.04	29.53±2.65	0.152
CD133^+^CD44^+^CD26^−^ subpopulation (×10^3^)	0.20±0.01	0.47±0.05	0.227	0.19±0.01	0.20±0.02	0.892
CD133^+^CD44^+^CD26^+^ subpopulation (×10^3^)	0.18±0.02	0.53±0.06	<0.001	0.16±0.02	0.20±0.02	0.503
CD133^+^CD44^−^CD26^+^ subpopulation (×10^3^)	0.84±0.19	2.70±0.56	0.028	0.16±0.02	0.19±0.02	0.590
CD133^+^CD44^+^CD54^−^ subpopulation (×10^3^)	0.11±0.02	0.11±0.01	0.857	0.09±0.01	0.14±0.02	0.195
CD133^+^CD44^+^CD54^+^ subpopulation (×10^3^)	0.31±0.03	0.91±0.11	<0.001	0.26±0.02	0.37±0.04	0.165
CD133^+^CD44^−^CD54+ subpopulation (×10^3^)	0.32±0.05	0.75±0.29	0.021	0.24±0.02	0.31±0.03	0.762
CD133^+^CD44^−^CD54^−^ subpopulation (×10^3^)	1.19±0.32	1.31±0.37	0.115	1.01±0.26	1.20±0.31	0.521

a CEA 0 <5 ng/ml, 1>5 to<20 ng/ml, 2>20 to <100 ng/ml, 3>100 ng/ml

b CA19-9 0 <20 ng/ml, 1>20 to<50 ng/ml, 2>50 to <200 ng/ml, 3>200 ng/ml

### Univariate and multivariate analysis of factors related to liver metastasis of CRC

To investigate the potential risk factors for CRC and liver metastasis, multivariable logistic regression analysis was performed using significant variables (Table 2 and Supplementary Table S3). Expression levels of serum CEA, CD133^+^CD44^+^CD54^+^ cellular subpopulation and extranodal tumor deposits showed predictive value for liver metastasis (*P*<0.05), and the odds ratios are 3.352 (95%C.I., 1.824–6.839), 2.898 (95%C.I.1.374–6.110), 25.820 (95%C.I.5.155–129.322), respectively. In addition, the expression levels of the CD133^+^CD44^+^CD26^+^ cellular subpopulation was also associated with the diagnosis of CRC (Supplementary Table S3). These data suggested that the CD133^+^CD44^+^CD54^+^ cellular subpopulation of peripheral blood was associated with liver metastasis of CRC thus has the potential of serving as novel predictor of liver metastasis.

**Table 2:** Univariate and multivariate logistic regression analysis of expression of cellular subpopulation of CTCs and clinical characteristics for liver metastasis

Variable	Univariate logistic analysis				Multivariate logistic analysis			
	Regression coefficient	*P* value	Odds ratio	Confidence interval	Regression coefficient	*P* value	Odds ratio	Confidence interval
Age	−0.38	0.058	0.963	0.926-1.001				
Serum CEA	1.252	<0.001	3.498	1.998-6.125	1.262	<0.001	3.532	1.824-6.839
Serum CA19-9	0.749	0.007	2.115	1.226-3.647				
Extra nodal tumor deposits	2.621	<0.001	13.750	4.508-41.938	3.251	<0.001	25.820	5.155-129.322
CD133^+^ subpopulation	<0.001	0.002	1.002	1.001-1.003				
CD26^+^ subpopulation	<0.001	0.031	1.001	1.000-1.002				
CD54^+^ subpopulation	0.001	0.010	1.001	1.000-1.002				
CD133^+^CD26^−^ subpopulation	<0.001	0.037	1.001	1.000-1.002				
CD133^+^CD26^+^ subpopulation	0.615	0.003	1.850	1.224-2.795				
CD133^−^CD26^+^ subpopulation	0.001	0.044	1.001	1.000-1.003				
CD133^+^CD44^+^ subpopulation	0.697	0.002	2.007	1.296-3.109				
CD133^−^CD44^+^ subpopulation	0.006	0.022	1.006	1.001-1.011				
CD133^+^CD54^−^ subpopulation	0.001	0.028	1.001	1.000-1.002				
CD133^+^CD54^+^ subpopulation	0.611	0.011	1.841	1.149-2.952				
CD133^−^CD54^+^ subpopulation	0.002	0.039	1.002	1.000-1.003				
CD133^+^CD44^+^CD26^+^ subpopulation	0.601	0.019	1.823	1.103-3.012				
CD133^+^CD44^−^CD26+ subpopulation	<0.001	0.075	1.000	1.000-1.001				
CD133^+^CD44^+^CD54^+^ subpopulation	0.645	0.002	1.907	1.259-2.888	1.064	0.005	2.898	1.374-6.110
CD133^+^CD44^−^CD54+ subpopulation	<0.001	0.005	1.001	1.000-1.002				

### CD133^+^CD44^+^CD54^+^ expression in peripheral blood may serve as a serum auxiliary diagnostic marker for liver metastasis of CRC

**Figure 5:**
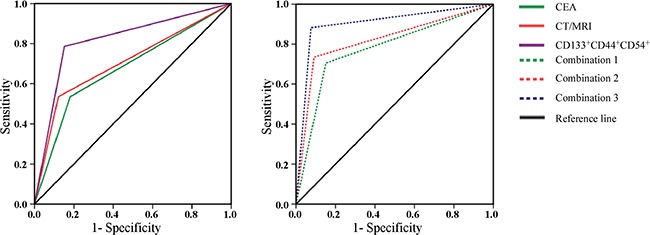
The receiver operating characteristics curves (ROC) and the corresponding values of area under the curve (AUC) of CD133^+^CD54^+^CD44^+^ cellular subpopulation of peripheral blood, CEA, CT/MRI or the combinations for CRC liver metastasis Left The ROC and AUC of CD133^+^CD54^+^CD44^+^ cellular subpopulation of peripheral blood, serum CEA level and CT/MRI for CRC liver metastasis. Right The ROC and AUC of CT/MRI combination with serum CEA level and/ or CD133^+^CD54^+^CD44^+^ cellular subpopulation of peripheral blood for CRC liver metastasis. The combination 1 (combination of abdomen CT/MRI with CEA), combination 2 (combination of abdomen CT/MRI with the CD133^+^CD44^+^CD54^+^ cellular subpopulation) and combination 3 (combination of abdomen CT/MRI with CEA and the CD133^+^CD44^+^CD54^+^ cellular subpopulation).

To validate the predictive ability of the CD133^+^CD44^+^54^+^ and CD133^+^CD44^+^26^+^ cellular subpopulations examined in peripheral blood, we calculated the ROC curve and the AUC for above 2 co-expression cellular subpopulation and compared with those of CEA and abdomen CT/MRI in different test groups. The sensitivity of the CD133^+^CD44^+^CD26^+^ cellular subpopulation was higher than serum CEA in both diagnosis of CRC and early-stage CRC (70.4% vs. 50.0% and 61.1% vs. 27.8%, respectively) (Table 3, Supplementary Figure S2, left panel). The specificity and the AUC of those two marker combinations were similar in both the diagnosis of CRC and early-stage CRC (Table 3, Supplementary Figure S2). Concerning liver metastasis of CRC, abdomen CT/MRI achieved 78.6% sensitivity and 84.8% specificity, significantly higher than CEA level and the CD133^+^CD44^+^CD54^+^ cellular subpopulation (53.6% and 81.8% for CEA, 71.0% and 75.4% for the CD133^+^CD44^+^CD54^+^ cellular subpopulation, respectively) (Table 3, Figure 5 left panel).

**Table 3:** Receiver operating characteristic curves (ROC) and the corresponding values of area under the curve (AUC) for CRC and CRC liver metastasis

Characteristic	Sensitivity (%)	Specificity (%)	AUC	Confidence interval
**CRC**				
CEA	50.0%	84.4%	0.625	0.517-0.733
CD133^+^CD44^+^CD54^+^	66.3%	75.0%	0.707	0.604-0.810
CD133^+^CD44^+^CD26^+^	70.4%	75.0%	0.727	0.625-0.829
**Early CRC**				
CEA	27.8%	84.4%	0.514	0.376-0.652
CD133^+^CD44^+^CD54^+^	58.3%	75.0%	0.667	0.537-0.797
CD133^+^CD44^+^CD26^+^	61.1%	75.0%	0.681	0.552-0.809
**CRC with liver metastasis**				
CEA	53.6%	81.8%	0.677	0.552-0.802
CD133^+^CD44^+^CD54^+^	71.0%	75.4%	0.707	0.583-0.832
CT/MRI	78.6%	84.8%	0.817	0.715-0.919
CT/MRI+CEA	70.6%	84.8%	0.777	0.674-0.881
CT/MRI+ CD133^+^CD44^+^CD54^+^	73.5%	90.9%	0.822	0.725-0.919
CT/MRI+CEA+CD133^+^CD44^+^CD54^+^	88.2%	92.4%	0.903	0.830-0.976

We next calculated the ROC and the AUC for the combinations of abdomen CT/MRI, CEA and CD133^+^CD44^+^CD54^+^ cellular subpopulation in samples with liver metastasis (CRC-LM1) and without (CRC-LM0). Combination 3, abdomen CT/MRI plus CEA and CD133^+^CD44^+^CD54^+^ cellular subpopulation, showed increased detection and discrimination rate, with a sensitivity of 88.2%and a specificity of 92.4%, much higher than that of Combination 1 (abdomen CT/MRI plus CEA, 70.6% and 84.8% respectively) and Combination 2 (abdomen CT/MRI plus CD133^+^CD44^+^CD54^+^ cellular subpopulation, 73.5% and 90.9% respectively). Thirty of 34 CRC-LM1 cases and 61 of 66 CRC-LM0 cases were correctly classified by combination 3, while four CRC-LM0 and five CRC-LM1 cases misclassified by combination 3. Most importantly, 37 patients with liver nodes could not be classified properly by single CT/MRI method. For these 37 patients, 15 of 19 CRC-LM1 cases and 13 of 18 CRC-LM0 cases were correctly classified by combination 3, while four CRC-LM0 cases and five CRC-LM1 cases misclassified. The AUC for Combination 3 was 0.903 (95% confidence interval 0.830-0.976) (Table 3, Figure 5 right panel). These data indicated that CD133^+^CD44^+^CD54^+^ cellular subpopulation of peripheral blood could be used as an auxiliary diagnostic marker for liver metastasis of CRC-LM improving the early detection of liver metastasis.

## DISCUSSION

Liver metastasis is the most important prognostic factor for CRC and the major cause of cancer-associated death in patients with CRC [1]. Therefore, the early and precise detection of liver metastasis is valuable in improving the overall prognosis of patients with CRC [2, 24-27]. The aim of this study was to investigate the cellular subpopulation of CTCs that may serve as a marker for the existence of CRC and/or CRC-LM.

We found that CD133, CD54, CD44 and CD26 is stably expressed in circulating cancer cells of the peripheral blood using FACS analysis and that the CD133^+^cellular subpopulation could be used as the baseline to select CTCs in patients with CRC due to its high expression. CD133 has been accepted as a cancer stem cell marker for colon cancer [28-30] and the expression of CD133 in the CRC primary tissue or liver metastases has been reported to be a significant prognostic factor [15,31,32]. Although antibody-mediated selection of EPCAM was still widely used as the CTC-enumerating techniques, several studies investigated the potential of CD133 as a cell surface marker in detection and isolation of CTCs. According to recent understanding, disseminating tumor cells may down-regulate their expression of epithelial-specific proteins via EMT transition and the EPCAM^−^ or EPCAM^low^ CTCs might be undetectable by EPCAM-based detection and isolation methods such as the CellSearch [10-12]. To circumvent these limitations, additional cell surface makers and marker-based CTC detection and enrichment platforms should be evaluated.

In this study, we observed that the expression of CTC subpopulations CD133^+^CD54^+^CD44^+^ and CD133^+^CD26^+^CD44^+^ were significantly higher in the peripheral blood of CRC-LM patients when compared with those without liver metastasis. Moreover, we also proved the association between CD133^+^CD44^+^CD54^+^ cellular subpopulation and the existence of CRC-LM through multivariate analysis. CD54 (intercellular adhesion molecule-1) is a member of the immunoglobulin super-family and is widely expressed in tumors, stroma and immune cells [33]. CD44 is one of the most frequently described markers of cancer initiating cells in numerous other malignancies and has been described as a signature of colon carcinoma initiating cells [14,16]. Even though, the present result is consistent with our previous finding in colorectal cancer initiating cells, the markers CD44 and CD54 have not yet been used to detect and isolate CTCs.

Cancer initiating cells (CICs) were identified as a rare cellular subpopulation with self-renewal, tumor-initiating, motile, invasive, heightened resistance to apoptosis, and instrumental to facilitating metastasis. However, CTCs possess the ability to reconstruct metastatic tumor that are similar to primary tumor and share the features of CICs [6,7,34,34]. CTCs held the capability of metastases were known as metastatic initiating cells (MICs). Previously, cellular subpopulations such as CD26^+^, CD133^+^CD44^+^, CD133^+^CXCR4^+^ had been sorted and identified as CICs or MICs [14,17,36]. The existence and phenotype of MICs had not been demonstrated in the peripheral blood until recently when the MICs were identified in the peripheral blood of primary human luminal breast cancer using a xenograft assay [37]. In this study, the CD133^+^CD54^+^CD44^+^ and CD133^+^CD26^+^CD44^+^cellular subpopulations were highly expressed in the peripheral blood of CRC-LM and multivariate analysis showed that the CD133^+^CD44^+^CD54^+^ cellular subpopulation was associated with liver metastasis. These findings share the similar biomarkers with MIC derived from breast cancer [37]. Thus, we hypothesized that the CD133^+^CD44^+^CD54^+^ cellular subpopulation is a fraction of CD44^+^CD54^+^ CICs located in the peripheral blood of patients with CRC. Further studies are needed to isolate the CD133^+^CD44^+^CD54^+^ cellular subpopulation from peripheral blood or tumor tissue of CRC patients, investigating the metastatic potential of this subpopulation by xenograft assay.

In patients with CRC, the early and precise detection of liver metastases offers the opportunity to perform liver-targeted therapy [2,26,27]. Nowadays, the general means of diagnosis is radioactive imaging with or without serum CEA and CA19-9 levels. The sensitivities of imaging methods for detecting liver metastasis range from 57% to–100% for ultrasound, 36%–99% for abdomen CT, 69%–96% for abdomen MRI according to current publications [27,38,39]. In our study, the sensitivity and specificity of CT/MRI was 78.6% and 84.8%, respectively. Meanwhile, the sensitivity of the CD133^+^CD44^+^CD54^+^ cellular subpopulation was higher than that of CEA (71.0%vs. 53.6%) for liver metastasis. These data indicated that CD133^+^CD44^+^CD54^+^ cellular subpopulation of peripheral blood could be used as an auxiliary diagnostic marker for liver metastasis.

The combination of abdomen CT/MRI with CD133^+^CD44^+^CD54^+^ cellular subpopulation and CEA showed increased detection and discrimination rate, and achieved the most satisfactory levels of sensitivity (88.2%) and specificity (92.4%). Furthermore, the marker combination is capable of discriminating metastasis from control samples, with an AUC equal to 0.903 (95% C.I.0.830–0.976). The CD133^+^CD44^+^CD54^+^ cellular subpopulation of peripheral blood may play a role in prediction of liver metastases and served as an auxiliary diagnosis marker. Current studies mainly focused on the clinical significance of CTCs as a prognostic or predictive factor [40-45] (predicting response to specific therapies). Although several studies demonstrated the diagnostic capabilities of metastatic proteins for CRC liver metastasis, our combination is more robust in both design and performance [46,47]. However, whether the CD133^+^CD44^+^CD54^+^cellular subpopulations of peripheral blood could be used as an auxiliary diagnosis marker for CRC and CRC-LM, or play a role in the postoperative follow-up needs further investigation.

In summary, we showed that the CD133^+^ cellular subpopulation could be used as the baseline to select and isolate CTCs in the peripheral blood of CRC patients through the FACS platform. Our studies identified that the expression of the CD133^+^CD54^+^CD44^+^cellular subpopulation of CTCs was significantly higher in the peripheral blood of CRC patient and was associated with liver metastasis. Furthermore, we found that the CD133^+^CD44^+^CD54^+^ cellular subpopulation of peripheral blood maybe used as an auxiliary diagnosis marker for liver metastasis. The molecular characterization and metastatic capacity of the CD133^+^CD44^+^CD54^+^ cellular subpopulation of peripheral blood warrants further investigation *in vitro* and *in vivo*.

## MATERIALS AND METHODS

### Sample collection

Peripheral blood samples were obtained from CRC patients at the Department of Gastrointestinal Surgery and from age-matched healthy individuals at the Department of Health Care Center, West China Hospital, Sichuan University, Guoxue Line 37, Chengdu, China. Primary CRC and hepatic metastatic samples were obtained from patients undergoing surgical resection at the Department of Gastrointestinal Surgery, West China Hospital, Sichuan University, Guoxue Line 37, Chengdu, China. Informed consent was obtained from all of the included individuals and the protocol was approved by the Institutional Review Board of West China Hospital, Sichuan University, Guoxue Line 37, Chengdu, China.

### Sample preparation

Peripheral blood samples were collected in a 5 ml Vacutainer tube containing EDTA as an anticoagulant and the CRC blood sample was collected before surgery or radiochemotherapy. All samples were shipped on ice to the laboratory and analyzed within 24 hours. Firstly, 4 ml of whole blood, 4 ml of red cell lysing/fixative solution and 32 ml of distilled water were mixed and incubated for 15 min. Red cell debris was washed out with two cycles of centrifugation (300 × *g* for 15 min and 10 min). After washing, phosphate buffered saline (PBS) was added to 200 μl of cell suspensions and added 4–10 μl each of fluorochrome labeled antibodies according to the protocol. The suspension was incubated in the room temperature for 30 min. The cells were then washed to remove excess reagents by centrifugation (300 × *g* for 10 min). After the final wash, 0.8 ml of PBS was added and the cell suspension was added to the FACS Calibur (BD Biosciences, San Jose, CA, USA).

Fresh tissue specimens from primary CRC, liver metastatic cancer and the comparative normal tissues were immediately minced on ice, suspended in the PBS and then shipped to the laboratory. Next, the tissue was gently minced and filtered (100 um) to remove large aggregates. The samples were then incubated for 60 min at 37 °C suspended in 50 ml of PBS containing 0.05% collagenase, with continuous stirring. The cell suspension was filtered (40 um) and then washed by centrifugation (300 × *g* for 15 min). After washing, PBS was added to the cells and incubated with fluorochrome labeled antibodies as described above.

### Flow cytometry analyses and sorting of CTCs

CTCs from cells suspension were characterized by multiparameter flow cytometry. The antibodies used in this study include: anti-human CD133-APC, CD44-FITC, CD44-APC-Cy7, CD54-Percp-cy5.5, CD54-PE, CD24-PE/Cy7, CD10-PECF594, CD26-PE, CD166-Percp-cy5.5, CD45-BV510, CD58-PE, CD66-PE, CD71-PE, CD117-PE, EPCAM-Percp-cy5.5, and EGFR-PE (all of the above-mentioned antibodies were purchased from BD Biosciences). DAPI was used to identify the dead cells. Evaluation of nucleated cells from whole cells suspensions was carried out using a FACS Canto Flow Cytometer (BD Biosciences) and data were analyzed using BD FACS Diva software. A range of internal quality assurance procedures was employed, including daily calibration of the optical alignment and fluidic stability of the flow cytometer using the seven-color Set-up Beads (BD Biosciences). The absolute CTCs or antibody-positive cell number was derived from the absolute number of the white blood cells provided by the hematological analyzer and percentage of CTCs or antibody-positive cell as determined by flow cytometry, using the following formula: percentage of cells × white blood cells count/100.

### Clinical information

All CRC patients were enrolled in the Department of Gastrointestinal Surgery, West China Hospital, Sichuan University, Guoxue Line 37, Chengdu, China from January 2014 to March 2015. All of the patients received an examination to determine the stage of cancer, including physical examination, colonoscopy, specimens histology, complete blood count, liver function, serum carcino-embryonic antigen (CEA), serum carbohydrate antigen 19-9 (CA19-9), thorax contrast-enhanced computed tomography (CT), abdomen contrast-enhanced CT or contrast-enhanced magnetic resonance imaging (MRI). The clinical T stage, lymph node metastasis and liver or lung metastases were made by the multidisciplinary teams though the iconographic examinations. The treatment decision for the patients, including surgical resection, preoperative chemotherapy, radiochemotherapy, palliation chemotherapy, radiochemotherapy, or palliation surgery was also made by the multidisciplinary teams.

### Statistical analyses

All of the experimental data were expressed as mean ± SD and statistically analyzed. The distribution of nominal- or ordinal-scaled variables was compared using the Pearson *x^2^* test. Cardinal variables were tested for normal distribution by the Kolmogorov–Smirnov test. Explorative comparison of independent groups was performed by the *t* test for normal distribution and the Mann–Whitney *U* test (two groups) or Kruskal–Wallis test (more than two groups) for the abnormal distributions. Univariate and multivariate analyses of potential metastatic predictive variables were carried out using the logistic regression model. Receiver operating characteristic (ROC) curves and the corresponding values of area under the curve (AUC) were generated to compare the predictive sensitivity and specificity. All statistical tests were performed two-sided, and P values less than 0.05 (*P*<0.05) were considered to be statistically significance. All statistical analyses were performed using SPSS Statistics Version 22 (SPSS Software, Inc., Chicago, IL, USA) and the GraphPad Prism 5 statistical software (GraphPad Software, Inc., San Diego, CA, USA).

## SUPPLEMENTARY MATERIALS FIGURES AND TABLES





## References

[1] Torre LA, Bray F, Siegel RL, Ferlay J, Lortet-Tieulent J, Jemal A (2012). Global cancer statistics. CA Cancer J Clin.

[2] van de Velde CJ, Boelens PG, Borras JM, Coebergh JW, Cervantes A, Blomqvist L, Beets-Tan RG, van den Broek CB, Brown G, Van Cutsem E, Espin E, Haustermans K, Glimelius B (2014). EURECCA colorectal: multidisciplinary management: European consensus conference colon & rectum. Eur J Cancer.

[3] Van Cutsem E, Nordlinger B, Adam R, Köhne CH, Pozzo C, Poston G, Ychou M, Rougier P (2006). Towards a pan-European consensus on the treatment of patients with colorectal liver metastases. Eur J Cancer.

[4] Hayashi M, Inoue Y, Komeda K, Shimizu T, Asakuma M, Hirokawa F, Miyamoto Y, Okuda J, Takeshita A, Shibayama Y, Tanigawa N (2010). Clinicopathological analysis of recurrence patterns and prognostic factors for survival after hepatectomy for colorectal liver metastasis. BMC Surg.

[5] Tsai MS, Su YH, Ho MC, Liang JT, Chen TP, Lai HS, Lee PH (2007). Clinicopathological features and prognosis in resectable synchronous and metachronous colorectal liver metastasis. Ann Surg Oncol.

[6] Yu M, Stott S, Toner M, Maheswaran S, Haber DA (2011). Circulating tumor cells: approaches to isolation and characterization. J Cell Biol.

[7] Pantel K, Alix-Panabieres C, Riethdorf S (2009). Cancer micrometastases. Nat Rev Clin Oncol.

[8] Kling J (2012). Beyond counting tumor cells. Nat Biotechnol.

[9] Attard G, Swennenhuis JF, Olmos D, Reid AH, Vickers E, A'Hern R, Levink R, Coumans F, Moreira J, Riisnaes R, Oommen NB, Hawche G, Jameson C (2009). Characterization of ERG, AR and PTEN gene status in circulating tumor cells from patients with castration-resistant prostate cancer. Cancer Res.

[10] Rhim AD, Mirek ET, Aiello NM, Maitra A, Bailey JM, McAllister F, Reichert M, Beatty GL, Rustgi AK, Vonderheide RH, Leach SD, Stanger BZ (2012). EMT and dissemination precede pancreatic tumor formation. Cell.

[11] Yu M, Bardia A, Wittner BS, Stott SL, Smas ME, Ting DT, Isakoff SJ, Ciciliano JC, Wells MN, Shah AM, Concannon KF, Donaldson MC, Sequist LV (2013). Circulating Breast Tumor Cells Exhibit Dynamic Changes in Epithelial and Mesenchymal Composition. Science.

[12] Chaffer CL, Weinberg RA (2011). A perspective on cancer cell metastasis. Science.

[13] Orfao A, Rodríguez C, Adansa JC, Ramos M, Gómez-Alonso A, Adansa JC, Rodríguez C, Orfao A (2005). Evaluation of Multiparameter Flow Cytometry for the Detection of Breast Cancer Tumor Cells in Blood Samples. American Journal of Clinical Pathology.

[14] Haraguchi N, Ohkuma M, Sakashita H, Matsuzaki S, Tanaka F, Mimori K, Kamohara Y, Inoue H, Mori M (2008). CD133+CD44+ population efficiently enriches colon cancer initiating cells. Ann Surg Oncol.

[15] Huang X, Sheng Y, Guan M (2012). Co-expression of stem cell genes CD133 and CD44 in colorectal cancers with early liver metastasis. Surg Oncol.

[16] Du L, Rao G, Wang H, Li B, Tian W, Cui J, He L, Laffin B, Tian X, Hao C, Liu H, Sun X, Zhu Y (2013). CD44-Positive Cancer Stem Cells Expressing Cellular Prion Protein Contribute to Metastatic Capacity in Colorectal Cancer. Cancer Research.

[17] Pang R, Law WL, Chu AC, Poon JT, Lam CS, Chow AK, Ng L, Cheung LW, Lan XR, Lan HY, Tan VP, Yau TC, Poon RT (2010). A subpopulation of CD26+ cancer stem cells with metastatic capacity in human colorectal cancer. Cell Stem Cell.

[18] De Chiara L, Rodriguez-Pineiro AM, Rodriguez-Berrocal FJ, Cordero OJ, Martínez-Ares D, Páez de la Cadena M (2010). Serum CD26 is related to histopathological polyp traits and behaves as a marker for colorectal cancer and advanced adenomas. BMC Cancer.

[19] Ahmed MA, Al-Attar A, Kim J, Watson NF, Scholefield JH, Durrant LG, Ilyas M (2009). CD24 shows early upregulation and nuclear expression but is not a prognostic marker in colorectal cancer. J Clin Pathol.

[20] Lugli A, Iezzi G, Hostettler I, Muraro MG, Mele V, Tornillo L, Carafa V, Spagnoli G, Terracciano L, Zlobec I (2010). Prognostic impact of the expression of putative cancer stem cell markers CD133, CD166, CD44s, EpCAM, and ALDH1 in colorectal cancer. Br J Cancer.

[21] Muraro MG, Mele V, Daster S, Han J, Heberer M, Cesare Spagnoli G, Iezzi G (2012). CD133+, CD166+CD44+, and CD24+CD44+ phenotypes fail to reliably identify cell populations with cancer stem cell functional features in established human colorectal cancer cell lines. Stem Cells Transl Med.

[22] Fan CW, Chen T, Shang YN, Gu YZ, Zhang SL, Lu R, OuYang SR, Zhou X, Li Y, Meng WT, Hu JK, Lu Y, Sun XF (2013). Cancer-initiating cells derived from human rectal adenocarcinoma tissues carry mesenchymal phenotypes and resist drug therapies. Cell Death and Disease.

[23] Chen T, Yang K, Yu J, Meng W, Yuan D, Bi F, Liu F, Liu J, Dai B, Chen X, Wang F, Zeng F, Xu H (2012). Identification and expansion of cancer stem cells in tumor tissues and peripheral blood derived from gastric adenocarcinoma patients. Cell Res.

[24] Pawlik TM, Scoggins CR, Zorzi D, Abdalla EK, Andres A, Eng C, Curley SA, Loyer EM, Muratore A, Mentha G, Capussotti L, Vauthey JN (2005). Effect of Surgical Margin Status on Survival and Site of Recurrence After Hepatic Resection for Colorectal Metastases. Annals of Surgery.

[25] Brouquet A, Vauthey JN, Contreras CM, Walsh GL, Vaporciyan AA, Swisher SG, Curley SA, Mehran RJ, Abdalla EK (2011). Improved survival after resection of liver and lung colorectal metastases compared with liver-only metastases: a study of 112 patients with limited lung metastatic disease. J Am Coll Surg.

[26] Marin C, Robles R, Lopez Conesa A, Torres J, Flores DP, Parrilla P (2013). Outcome of strict patient selection for surgical treatment of hepatic and pulmonary metastases from colorectal cancer. Dis Colon Rectum.

[27] Kinkel K, Lu Y, Both M, Warren RS, Thoeni RF (2002). Detection of hepatic metastases from cancers of the gastrointestinal tract by using noninvasive imaging methods (US, CT, MR imaging, PET) a meta-analysis. Radiology.

[28] Miraglia S, Godfrey W, Yin AH, Atkins K, Warnke R, Holden JT, Bray RA, Waller EK, Buck DW (1997). A Novel Five-Transmembrane Hematopoietic Stem Cell Antigen Isolation, Characterization, and Molecular Cloning. Blood.

[29] Ricci-Vitiani L, Lombardi DG, Pilozzi E, Biffoni M, Todaro M, Peschle C, De Maria R (2007). Identification and expansion of human colon-cancer-initiating cells. Nature.

[30] O'Brien CA, Pollett A, Gallinger S, Dick JE (2007). A human colon cancer cell capable of initiating tumour growth in immunodeficient mice. Nature.

[31] Maeda S, Shinchi H, Kurahara H, Mataki Y, Maemura K, Sato M, Natsugoe S, Aikou T, Takao S (2008). CD133 expression is correlated with lymph node metastasis and vascular endothelial growth factor-C expression in pancreatic cancer. Br J Cancer.

[32] Piscuoglio S, Lehmann FS, Zlobec I, Tornillo L, Dietmaier W, Hartmann A, Wünsch PH, Sessa F, Rümmele P, Baumhoer D, Terracciano LM (2011). Effect of EpCAM, CD44, CD133 and CD166 expression on patient survival in tumours of the ampulla of Vater. Journal of Clinical Pathology.

[33] Ieda J, Yokoyama S, Tamura K, Takifuji K, Hotta T, Matsuda K, Oku Y, Nasu T, Kiriyama S, Yamamoto N, Nakamura Y, Shively JE, Yamaue H (2011). Re-expression of CEACAM1 long cytoplasmic domain isoform is associated with invasion and migration of colorectal cancer. Int J Cancer.

[34] Mani SA, Guo W, Liao MJ, Eaton EN, Ayyanan A, Zhou AY, Brooks M, Reinhard F, Zhang CC, Shipitsin M, Campbell LL, Polyak K, Brisken C (2008). The epithelial-mesenchymal transition generates cells with properties of stem cells. Cell.

[35] Plaks V, Koopman CD, Werb Z (2013). Cancer. Circulating tumor cells. Science.

[36] Zhang SS, Han ZP, Jing YY, Tao SF, Li TJ, Wang H, Wang Y, Li R, Yang Y, Zhao X, Xu XD, Yu ED, Rui YC (2012). CD133(+)CXCR4(+) colon cancer cells exhibit metastatic potential and predict poor prognosis of patients. BMC Med.

[37] Baccelli I, Schneeweiss A, Riethdorf S, Stenzinger A, Schillert A, Vogel V, Klein C, Saini M, Bäuerle T, Wallwiener M, Holland-Letz T, Höfner T, Sprick M (2013). Identification of a population of blood circulating tumor cells from breast cancer patients that initiates metastasis in a xenograft assay. Nat Biotechnol.

[38] Floriani I, Torri V, Rulli E, Garavaglia D, Compagnoni A, Salvolini L, Giovagnoni A (2010). Performance of imaging modalities in diagnosis of liver metastases from colorectal cancer: a systematic review and meta-analysis. J Magn Reson Imaging.

[39] KA P, N B (2008). Current diagnostic and therapeutic approaches for colorectal cancer liver metastasis. HIPPOKRATIA.

[40] Iinuma H, Watanabe T, Mimori K, Adachi M, Hayashi N, Tamura J, Matsuda K, Fukushima R, Okinaga K, Sasako M, Mori M (2011). Clinical significance of circulating tumor cells, including cancer stem-like cells, in peripheral blood for recurrence and prognosis in patients with Dukes' stage B and C colorectal cancer. J Clin Oncol.

[41] Cohen SJ, Punt CJ, Iannotti N, Saidman BH, Sabbath KD, Gabrail NY, Picus J, Morse MA, Mitchell E, Miller MC, Doyle GV, Tissing H, Terstappen LW (2009). Prognostic significance of circulating tumor cells in patients with metastatic colorectal cancer. Ann Oncol.

[42] Sastre J, Maestro ML, Gomez-Espana A, Rivera F, Valladares M, Massuti B, Benavides M, Gallén M, Marcuello E, Abad A, Arrivi A, Fernández-Martos C, González E (2012). Circulating tumor cell count is a prognostic factor in metastatic colorectal cancer patients receiving first-line chemotherapy plus bevacizumab: a Spanish Cooperative Group for the Treatment of Digestive Tumors study. Oncologist.

[43] Gazzaniga P, Gianni W, Raimondi C, Gradilone A, Lo Russo G, Longo F, Gandini O, Tomao S, Frati L (2013). Circulating tumor cells in high-risk nonmetastatic colorectal cancer. Tumour Biol.

[44] Katsuno H, Zacharakis E, Aziz O, Gradilone A, Lo Russo G, Longo F, Gandini O, Tomao S, Frati L (2008). Does the presence of circulating tumor cells in the venous drainage of curative colorectal cancer resections determine prognosis? A meta-analysis. Ann Surg Oncol.

[45] Tol J, Koopman M, Miller MC, Tibbe A, Cats A, Creemers GJ, Vos AH, Nagtegaal ID, Terstappen LW, Punt CJ (2010). Circulating tumour cells early predict progression-free and overall survival in advanced colorectal cancer patients treated with chemotherapy and targeted agents. Ann Oncol.

[46] Sun L, Pan J, Peng L, Fang L, Zhao X, Sun L, Yang Z, Ran Y (2012). Combination of haptoglobin and osteopontin could predict colorectal cancer hepatic metastasis. Ann Surg Oncol.

[47] Ryu HS, Kim WH, Ahn S, Kim DW, Kang SB, Park HJ, Park YS, Lee CH, Lee HS (2014). Combined morphologic and molecular classification for predicting lymph node metastasis in early-stage colorectal adenocarcinoma. Ann Surg Oncol.

