# Supplementary material for: CD133+CD54+CD44+ circulating tumor cells as a biomarker of treatment selection and liver metastasis in patients with colorectal cancer
Source: Oncotarget. 2016 Oct 15;7(47):77389–403. doi: 10.18632/oncotarget.12675 (PMC5363593; doi:10.18632/oncotarget.12675)
Supplement: Supplementary file 1 [file oncotarget-07-77389-s001.pdf]

CD133⁺CD54⁺CD44⁺ circulating tumor cells as a biomarker of treatment selection and liver metastasis in patients with colorectal cancer

SUPPLEMENTARY FIGURES AND TABLES

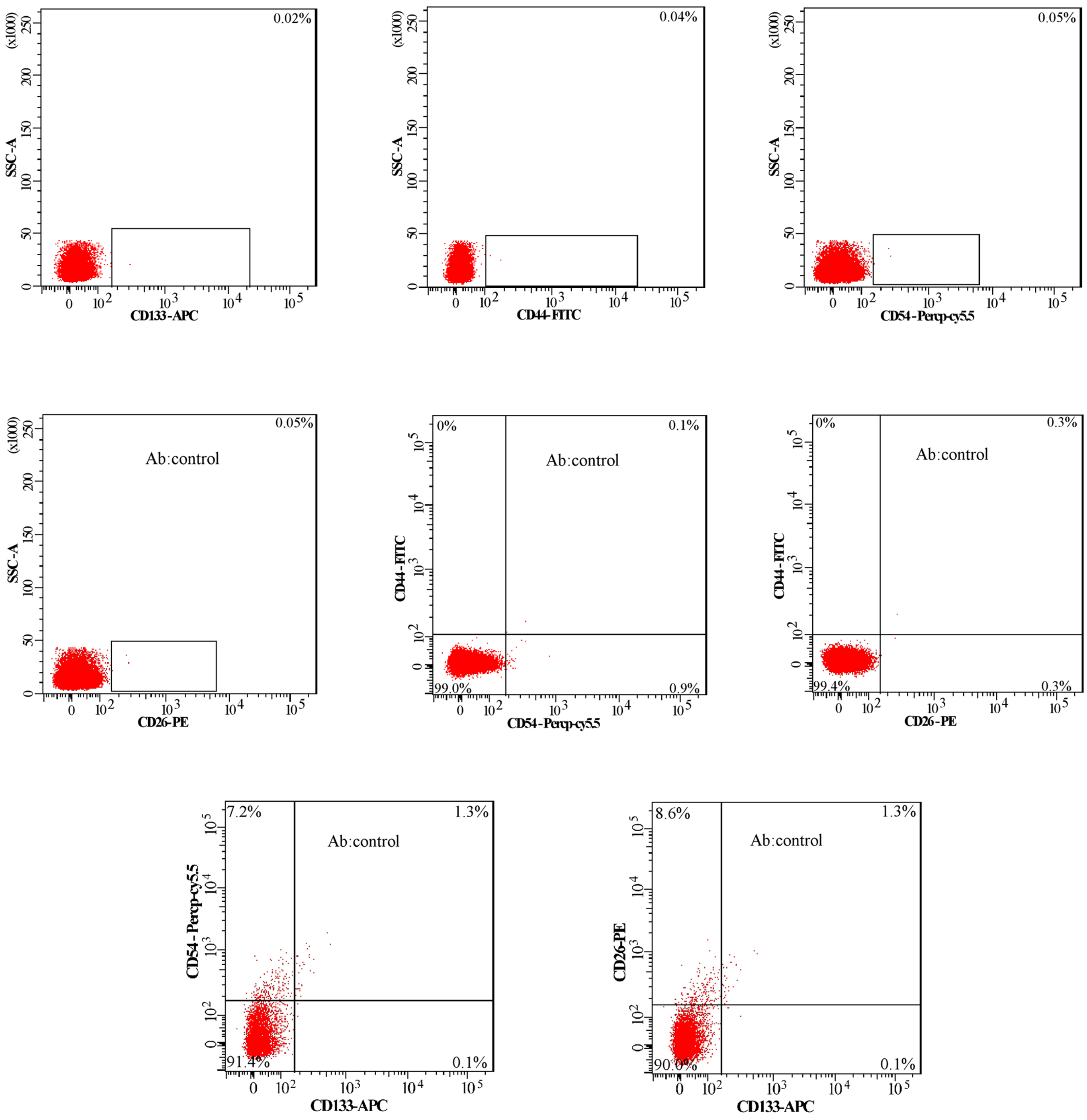

Supplementary Figure S1: The AB control for each marker respectively or federatively.

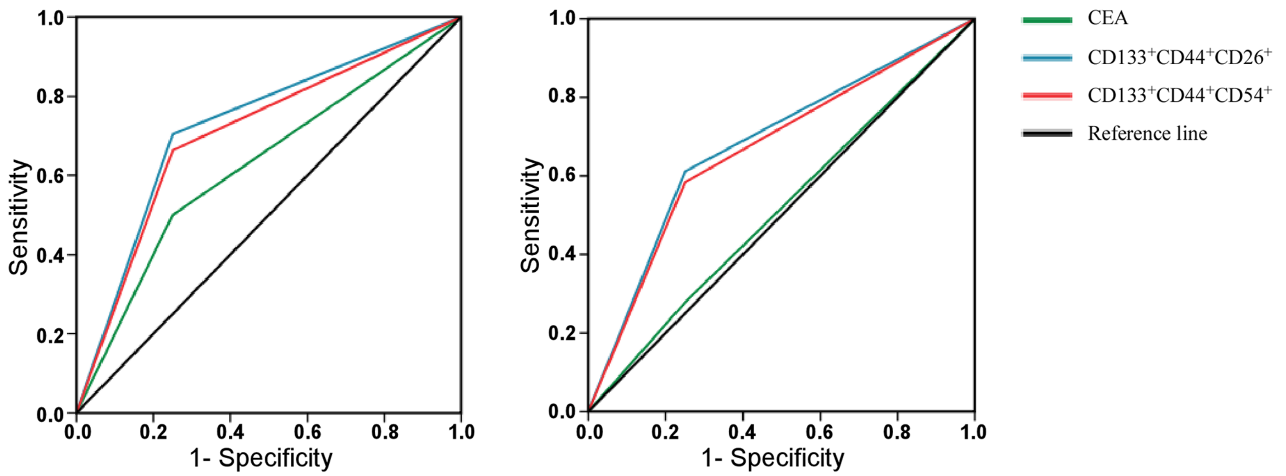

Supplementary Figure S2: The receiver operating characteristics curves (ROC) and the corresponding values of area under the curve (AUC) of CD133$^+$CD54$^+$CD44$^+$ and CD133$^+$CD26$^+$CD44$^+$cellular subpopulation of peripheral blood, CEA for CRC and early CRC. Left The ROC and AUC of CD133$^+$CD54$^+$CD44$^+$ and CD133$^+$CD26$^+$CD44$^+$ cellular subpopulation of peripheral blood and serum CEA level for CRC. Right The ROC and AUC of CD133$^+$CD54$^+$CD44$^+$ and CD133$^+$CD26$^+$CD44$^+$ cellular subpopulation of peripheral blood and serum CEA level for early CRC.

Supplementary Table S1: Expression of potential circulating cancer cell markers in peripheral blood between 10 pairs age-matched CRC and health individual (Test 1)

markers	CRC patients (×10^3)	Health individual (×10^3)	*P* value
CD10	0.32±0.08	0.29±0.09	0.516
CD24	23.31±15.23	15.78±10.12	0.308
CD26	33.91±3.28	32.47±4.21	0.414
CD44	218.35±29.26	151.29±19.17	0.376
CD54	109.31±14.78	55.77±5.12	0.051
CD58	14.97±5.81	15.13±6.02	0.950
CD66	2.31±1.76	2.27±1.81	0.990
CD71	0.84±0.45	0.91±0.51	0.972
CD117	0.19±0.12	0.31±0.14	0.069
CD133	2.72±0.41	0.79±0.17	0.008
CD166	25.76±16.32	15.13±11.25	0.127
CD326	129.16±17.49	123.68±18.25	0.532
EGFR	0.07±0.02	0.05±0.03	0.151

Supplementary Table S2: Cellular subpopulations of CTCs between health individuals and CRC.

See Supplementary File 1

Supplementary Table S3: Univariate and multivariate logistic regression analysis of expression of cellular subpopulation of CTCs for colorectal cancer

Variable	Univariate logistic analysis				Multivariate logistic analysis			
	Regression coefficient	*P* value	Odds ratio	Confidence interval	Regression coefficient	*P* value	Odds ratio	Confidence interval
$CD133^+$ subpopulation	0.001	<0.001	1.001	1.001-1.002				
$CD133^+CD44^+CD26^+$ subpopulation	1.211	<0.001	3.357	1.843-6.114	0.844	0.019	2.326	1.146-4.719
$CD133^+CD44^+CD54^-$ subpopulation	0.005	0.088	1.005	0.999-1.010				
$CD133^+CD44^+CD54^+$ subpopulation	0.988	<0.001	2.688	1.614-4.472				
$CD133^+CD44^-$ CD54+ subpopulation	0.002	0.002	1.002	1.001-1.003				
$CD133^+CD44^-CD54^-$ subpopulation	0.002	0.062	1.002	0.999-1.003				
