# Supplementary material for: CD133+CD54+CD44+ circulating tumor cells as a biomarker of treatment selection and liver metastasis in patients with colorectal cancer
Source: Oncotarget. 2016 Oct 15;7(47):77389–403. doi: 10.18632/oncotarget.12675 (PMC5363593; doi:10.18632/oncotarget.12675)
Supplement: Supplementary file 2 [file oncotarget-07-77389-s002.docx]

Supplementary Table 2. Cellular subpopulations of CTCs between health individuals and CRC

|  | Health individual | CRC | *P* value | Health individual | Early CRC | *P* value |
| --- | --- | --- | --- | --- | --- | --- |
| Gender (male: female) | 21:12 | 62:38 | 0.912 | 21:12 | 24:12 | 0.873 |
| Age (years) | 61.19±11.37 | 62.15±10.13 | 0.275 | 61.19±11.37 | 63.78±11.85 | 0.198 |
| CD133^+^ subpopulation (×10^3^) | 1.05±0.05 | 3.92±0.46 | <0.001 | 1.05±0.05 | 2.78±0.26 | <0.001 |
| CD54^+^ subpopulation (×10^3^) | 44.34±9.81 | 103.31±16.57 | 0.046 | 44.34±9.81 | 42.97±11.26 | 0.915 |
| CD26^+^ subpopulation (×10^3^) | 83.60±11.59 | 118.70±25.18 | 0.479 | 83.60±11.59 | 50.29±8.61 | 0.204 |
| CD44^+^ subpopulation (×10^3^) | 150.86±18.87 | 207.83±30.08 | 0.399 | 150.86±18.87 | 95.50±14.04 | 0.169 |
| CD133^+^CD44^-^ subpopulation (×10^3^) | 0.66±0.19 | 2.08±0.35 | 0.396 | 0.66±0.19 | 0.99±0.13 | 0.181 |
| CD133^+^CD44^+^ subpopulation(×10^3^) | 0.31±0.05 | 0.74±0.07 | 0.385 | 0.31±0.05 | 0.54±0.12 | 0.561 |
| CD133^-^CD44^+^ subpopulation (×10^3^) | 6.57±0.56 | 10.66±1.72 | 0.184 | 6.57±0.56 | 6.99±0.86 | 0.810 |
| CD133^+^CD54^-^ subpopulation (×10^3^) | 0.73±0.08 | 1.26±0.18 | 0.108 | 0.73±0.08 | 1.16±0.19 | 0.451 |
| CD133^+^CD54^+^ subpopulation (×10^3^) | 0.61±0.06 | 1.01±0.13 | 0.107 | 0.61±0.06 | 0.58±0.14 | 0.847 |
| CD133^-^CD54^+^ subpopulation (×10^3^) | 1.33±0.15 | 2.04±0.34 | 0.248 | 1.33±0.15 | 1.66±0.21 | 0.468 |
| CD133^+^CD26^-^ subpopulation (×10^3^) | 0.24±0.02 | 0.32±0.03 | 0.300 | 0.24±0.02 | 0.26±0.03 | 0.820 |
| CD133^+^CD26^+^ subpopulation (×10^3^) | 0.26±0.02 | 0.74±0.19 | 0.191 | 0.26±0.02 | 0.45±0.12 | 0.420 |
| CD133^-^CD26^+^ subpopulation (×10^3^) | 80.61±11.98 | 106.91±23.09 | 0.659 | 80.61±11.98 | 43.27±7.58 | 0.092 |
| CD26^+^CD44^-^ subpopulation (×10^3^) | 35.31±6.19 | 56.26±9.19 | 0.361 | 35.31±6.12 | 28.86±5.03 | 0.447 |
| CD26^+^CD44^+^ subpopulation (×10^3^) | 35.65±6.83 | 47.42±8,23 | 0.621 | 35.65±6.98 | 17.38±3.65 | 0.102 |
| CD26^-^CD44^+^ subpopulation (×10^3^) | 4.04±0.89 | 7.27±1.27 | 0.215 | 4.04±0.89 | 4.87±1.15 | 0.758 |
| CD54^+^CD44^-^ subpopulation (×10^3^) | 106.76±14.70 | 123.22±23.53 | 0.706 | 106.76±14.70 | 65.86±9.87 | 0.176 |
| CD54^+^CD44^+^ subpopulation (×10^3^) | 45.87±5.60 | 85.64±15.22 | 0.145 | 45.87±5.60 | 33.49±4.59 | 0.317 |
| CD54^-^CD44^+^subpopulation (×10^3^) | 20.71±2.55 | 31.48±4.09 | 0.157 | 20.71±2.55 | 19.24±3.04 | 0.830 |
| CD133^+^CD44^+^CD26^-^ subpopulation (×10^3^) | 0.18±0.02 | 0.21±0.02 | 0.393 | 0.18±0.02 | 0.19±0.01 | 0.716 |
| CD133^+^CD44^+^CD26^+^ subpopulation (×10^3^) | 0.06±0.01 | 0.28±0.04 | 0.003 | 0.06±0.01 | 0.16±0.02 | 0.005 |
| CD133^+^CD44^-^CD26^+^ subpopulation (×10^3^) | 0.11±0.01 | 1.38±0.04 | 0.059 | 0.11±0.01 | 0.97±0.26 | 0.082 |
| CD133^+^CD44^+^CD54^-^ subpopulation (×10^3^) | 0.07±0.01 | 0.11±0.02 | 0.076 | 0.07±0.01 | 0.09±0.01 | 0.258 |
| CD133^+^CD44^+^CD54^+^ subpopulation (×10^3^) | 0.14±0.02 | 0.51±0.07 | 0.005 | 0.14±0.02 | 0.26±0.02 | 0. 042 |
| CD133^+^CD44^-^CD54+ subpopulation (×10^3^) | 0.26±0.02 | 0.41±0.06 | 0.005 | 0.26±0.02 | 0.24±0.02 | 0. 670 |
| CD133^+^CD44^-^CD54^-^ subpopulation (×10^3^) | 0.48±0.04 | 1.61±0.18 | 0.108 | 0.48±0.04 | 1.01±0.26 | 0.089 |
